# Melatonin Prevents Mice Cortical Astrocytes From Hemin-Induced Toxicity Through Activating PKCα/Nrf2/HO-1 Signaling *in vitro*

**DOI:** 10.3389/fnins.2019.00760

**Published:** 2019-07-25

**Authors:** Xiao Chen, Zhiyu Xi, Huaibin Liang, Yuhao Sun, Zhihong Zhong, Baofeng Wang, Liuguan Bian, Qingfang Sun

**Affiliations:** ^1^Department of Neurosurgery, Ruijin Hospital, School of Medicine, Shanghai Jiao Tong University, Shanghai, China; ^2^Department of Neurology, Ruijin Hospital, School of Medicine, Shanghai Jiao Tong University, Shanghai, China; ^3^Department of Neurosurgery, Ruijin Hospital Luwan Branch, School of Medicine, Shanghai Jiao Tong University, Shanghai, China

**Keywords:** intracerebral hemorrhage, hemin, melatonin, PKCα, Nrf2, oxidative stress

## Abstract

Secondary injuries mediated by oxidative stress lead to deterioration of neurological functions after intracerebral hemorrhage (ICH). Cortical astrocytes are among the most important cells in the central nervous system (CNS), and play key roles in maintaining redox homeostasis by providing oxidative stress defense. Hemin is a product of hemoglobin degradation, which has strong toxicity and can induce reactive oxygen species (ROS). Melatonin (Mel) and its metabolites are well tolerated without toxicity, prevent tissue damage as well as effectively assist in scavenging free radicals. We evaluated the hemin neurotoxicity to astrocytes and the resistance of Mel-treated astrocytes to hemin neurotoxicity. And we found Mel induced PKCα phosphorylation (p-PKC), nuclear translocation of Nrf2 in astrocytes, and upregulation of HO-1, which contributed to the reduction of ROS accumulation and cell apoptosis. Nrf2 and HO1 protein expression upregulated by Mel were decreased after administration of PKC inhibitor, Ro 31-8220 (Ro 31). Luzindole (Luz), a melatonin receptor inhibitor, suppressed p-PKCα, HO-1, and Nrf2 expression upregulated by Mel and increased cell apoptosis rate. The upregulation of HO-1 induced by Mel was depressed by knocking down Nrf2 expression by siRNA, which also decreased the resistance of astrocytes to toxicity of hemin. Mel activates astrocytes through PKCα/Nrf2/HO-1 signaling pathway to acquire resistance to toxicity of hemin and resist from oxidative stress and apoptosis. The positive effect of Mel on PKCα/Nrf2/HO-1 signaling pathway may become a new target for neuroprotection after intracerebral hemorrhage.

## Introduction

ICH is a particularly destructive form of stroke with high mortality and morbidity, and survivors typically have severe nervous harm (Qureshi et al., 2009; Keep et al., 2012). Although surgical decompression of hemorrhage is widely believed to be a life-saving method, there is no authenticated medical or surgical treatment for ICH (Adeoye and Broderick, 2010; Keep et al., 2012; Hemphill et al., 2015). Mounting evidence suggests that intracerebral infusion of hemoglobin (Hb) and its catabolite such as iron, bilirubin and hemin is a major cause of brain injury induced by ICH (Zhao et al., 2011; Keep et al., 2012; Xi et al., 2014). These molecules increase the secretion of inflammatory cytokines including IL-1β and TNF-α, which play a key role in inflammation and enlarge the inflammatory cascade (Wang J. et al., 2018). Oxidative stress is a state in which reactive oxygen species (ROS) production and antioxidant capacity are imbalanced due to the dysfunction of the cellular antioxidant system (Santofimia-Castano et al., 2015). Excessive ROS could lead to oxidative stress, destroying DNA, lipids and protein, and ultimately leading to irreversible damage and apoptosis of cells (Jung et al., 2010; Reczek and Chandel, 2015). The astrocyte, the major gliocyte in CNS, helps to maintain CNS stability and protects neurons against oxidative stress, besides providing neurotrophic factors (Huang et al., 2016; Liu et al., 2017; Wu et al., 2017). Therefore, inhibition of oxidative stress in astrocytes is paramount.

Protein kinase C (PKC) enzymes play a major role in many metabolic and signaling pathways, and participate in the regulation of gene expression, cell growth, migration, proliferation, differentiation and apoptosis. Therefore, lack of PKC and/or its dysregulation may lead to different pathologies, such as diabetes, heart failure, Alzheimer’s and Parkinson’s diseases, inflammatory diseases, oxidative stress, and even cancer (Isakov, 2018). PKCα is a typical subtype of PKC and plays an important role in antioxidant stress (Chueakula et al., 2018). It has been reported that phosphorylation of Nrf2 by PKC is a key event for Nrf2 nuclear translocation in response to oxidative stress (Huang et al., 2000, 2002). Nrf2 is a member of NF-E2 family of nuclear basic leucine zipper transcription factors, being a key transcription factor that regulates antioxidant reaction against ROS (Itoh et al., 1999; Shih et al., 2003). Nrf2 is usually combined with an actin binding protein Kelch-like ECH associated protein 1 (Keap1) and anchored in the cytoplasm (Jung et al., 2010; Negi et al., 2011; Wang et al., 2012; Deng et al., 2015). Upon cells stimulation, Nrf2 then escapes from Keap1-mediated degradation, transfers from cytosol into nucleus, and subsequently binds to a promoter sequence called antioxidant response (ARE) (Tao et al., 2013; Kleszczynski et al., 2016) to produce a cytoprotective response characterized by high expression of antioxidant enzymes such as hemeoxygenase-1 (HO-1), NAD(P)H quinone oxidoreductase 1 (NQO1), Superoxide Dismutase 2 (SOD2), glutamate cysteine ligase (GCL), and glutathione S-transferase (GST), for example (Kensler et al., 2007; Liu et al., 2015; Kleszczynski et al., 2016). Among them, HO-1 presents a cytoprotective effect on oxidative and inflammatory stress, showing an important metabolic function. It is also the rate-limiting step of oxidative catabolism in heme group (Parada et al., 2014). In different cellular models, the induction of HO-1 is usually related to cell protection, including cerebral ischemia (Parada et al., 2014; Liu et al., 2015).

Melatonin (N-acetyl-5-methoxytryptamine, Mel) is a neurohormone produced in the pineal gland and released in the blood and cerebrospinal fluid (CSF) in a circadian rhythm (Aladag et al., 2009; Cao et al., 2017; Cipolla-Neto and Amaral, 2018). Mel as well as its metabolites are well tolerated without toxicity, prevent tissue damage as well as effectively assist in scavenging hydroxyl radical (HO), nitric oxide (NO), superoxide anion radical (O2 -), peroxynitrite anion (ONOO–), and peroxynitrous acid (ONOOH), and other free radicals (Reiter et al., 2000; Wu et al., 2012). Recently, the effects of Mel on Nrf2 pathway have attracted more attention, specially due to its neuroprotective effect (Negi et al., 2011; Wang et al., 2012; Deng et al., 2015; Kleszczynski et al., 2016; Trivedi et al., 2016; Cao et al., 2017). Mel has already been reported to decrease neuroinflammation and oxidative stress via Nrf2 in experimental diabetic neuropathy (Negi et al., 2011). Wang et al. (2012) have evaluated the protection of Mel on early brain injury from subarachnoid hemorrhage (SAH) via the Nrf2-ARE pathway.

Whether the anti-oxidative effect of Mel in hemin treated astrocytes is related to the PKCα/Nrf2/HO-1 signaling pathway has not been thoroughly studied. Consequently, our team assumed Mel regulated the signaling pathway of PKCα/Nrf2/HO-1 and might be an effective way to combat oxidative damage induced by hemin. In our study, we evaluated cell viability and apoptosis of Mel-treated astrocytes exposed to hemin. ROS, TNNEL staining, immunostaining, and protein expression of PKCα, Nrf2 and HO-1 were evaluated to study the resistance mechanisms of Mel-treated astrocytes to Hemin oxidative stress through PKCα/Nrf2/HO-1 signaling pathway.

## Materials and Methods

### Isolation and Culture of Astrocytes

All experimental schemes were authorized by the Institutional Animal Care and Use Committee of Shanghai Jiao Tong University in Shanghai, China. The primary astrocyte cells were prepared from pallium of newborn C57BL/6 mice, within 24 h from birth, obtained from Jester Laboratory Animal Co., Ltd. (Shanghai, China). After removing meninges and blood vessels as much as possible, the remaining cortical tissues were gently ground with 0.25% trypsin and digested at 37°C for 10 min, then plated on a 75 cm^2^ flask coated with poly-D-lysine (Corning, United States) at a density of 20,000 cells/cm^2^, and kept at 37°C at 95% humidity and 5% carbon dioxide (CO_2_). The cells fused in 13–14 days, and half of the media was replaced by fresh media every 4 days. Pure second-to eighth-generation astrocytes were used for the following experiments.

### Study Design

The study was performed in three parts. *In vitro* experiments were designed as follows. In the first part, we evaluated the hemin neurotoxicity to astrocytes and the resistance of Mel-treated astrocytes to hemin neurotoxicity. In the second part, we specifically focused on PKC inhibitor, Ro 31-8220 (Ro 31) and the Mel receptor inhibitor, Luzindole (Luz). The regulation of Ro 31 and Luz on hemin resistance in Mel-treated astrocytes was studied in this part. In the third part, we studied whether Mel-treated astrocytes transfected with 50 nM si-Nrf2 could resist the hemin neurotoxicity.

#### Experiment 1

Astrocytes were seeded on 6, 12, 24, or 96-well plates, treated with 30 μM hemin for 24 h, with or without 30 or 60 μM Mel (Control, Control + 60 μM Mel, Hemin, Hemin + 30 μM Mel, Hemin + 60 μM Mel). Dosages of hemin and Mel were chosen as described previously (Wang Z. et al., 2018). Cells were gathered for cell viability assay, luciferase reporter gene assay, TUNEL staining, intracellular ROS detection, immunostaining, western blotting analysis, and real-time PCR analysis.

#### Experiment 2

Astrocytes were seeded on 6, 12, or 24-well plates, pre-treated with or without 1 μM Luz/3 μM Ro 31 for 6 h, then exposed to 30 μM hemin, with or without 60 μM Mel for 24 h (Control, Hemin, Hemin + Mel, Hemin + Mel + Luz/ Ro 31). The dosage of Ro 31 and Luz was selected according to previous studies (Juszczak et al., 2014; Santofimia-Castano et al., 2015). Cells were gathered for TUNEL staining, immunostaining and western blotting analysis.

#### Experiment 3

Fifty nanomolar si-Nrf2 or negative control siRNA (si-NC) were transfected to astrocytes for 48 h, followed by 30 μM hemin incubation, with or without 60 μM Mel for 24 h (Control, Hemin + Mel, Hemin + si-NC + Mel, Hemin + si-Nrf2 + Mel). Cells were gathered and operated according to methods of experiment 2.

### Drug Administration and siRNA Transfection

Hemin and Mel (Aladdin, China) were dissolved in absolute ethyl alcohol and diluted with 0.9% normal saline. Ro 31 were purchased from TargetMol, United States. Luz were purchased from Santa Cruz Biotechnology, United States. We transfected astrocytes with Nrf2 specific small interfering RNA (si-Nrf2) (GenePharma, China) by Lipofectamine^®^ 2000 transfection reagent (Invitrogen, United States) according to the manufacturer’s instructions. Western blotting was applied to prove the si-Nrf2 knockdown efficiency.

### Cell Viability Assay

Cell viability was assessed using Cell Counting Kit-8 (CCK-8) (Beyotime, China) according to the manufacturer’s instructions. Cells were seeded into a 96-well plate at a density of 10^4^ per well. After 24 h, the cells were treated with 0, 5, 10, 20, 30, 40, and 50 μM hemin with or without 60 μM Mel for 24 h. Then 10 μL CCK-8 working fluid was added to each pore and cultured for 4h at cell culture incubator with 37°C, 95% humidity and 5% CO_2_. The results of CCK-8 was tested by the microplate reader (Biotec, United States) at 450 nm and expressed as a percentage of the control group.

### Cytotoxicity Assessment by Lactate Dehydrogenase (LDH) Assay

Lactate dehydrogenase cytotoxicity kit (Beyotime Biotechnology, China) was used to detected cytotoxicity according to the manufacturer’s instructions. Cells were plated in 24-well plates. Cells were exposed to 30 μM hemin with or without 30 or 60 μM Mel for 24 h. The results of LDH was tested by the microplate reader (Biotec, United States) at 490 nm and expressed as a percentage of the control group.

### Detection of ROS

Cells were plated on 6-well plates, handled according to experiment design. ROS assay kit (Beyotime Biotechnology, China) was used to detect ROS accumulation according to the manufacturer’s instructions. Astrocytes were incubated with 10 μM DCFH-DA in serum free DMEM for 30 min at cell culture incubator with 37°C under 95% humidity with 5% CO2. Cells were washed with phosphate buffer solution (PBS) three times, and five randomly fields were pictured using a fluorescence microscope (Leica, Germany).

### Plasmid Constructs and Luciferase Reporter Gene Assay

Luciferase reporter gene assay was used to analyze whether there was a direct link between Nrf2 and HO-1 in primary astrocytes. A fragment of HO-1 that contains the promotor binding sequence (−500 bp upstream to 100 bp downstream) was cloned into a luciferase reporter construct (GenePharma). Overexpressed Nrf2 plasmid (Nrf2) (GenePharma) was constructed using the empty vector PCDNA 3.1 and transfected into primary astrocytes on 12-well plates. PCDNA 3.1 was used as a negative control (NC). Luciferase activity was measured 24 h after transfection using Dual-Glo Luciferase Reporter Assay kit (Promega) according to the directions of the manufacturer. Experiments were repeated three times independently.

### TUNEL Staining

TUNEL staining (*In Situ* Cell Death Detection Kit, Roche, Germany) was used to detected cellular apoptosis. Astrocytes were seeded onto coverslips and handled according to experiment designs. Then the cells were fixed in 4% paraformaldehyde (PFA) for 10 min. After washing with PBS three times, cells were permeated with 0.3% Triton X-100 for 10 min. The coverslips were embedded under the reaction fluid in dark humidified atmosphere for 60 min at 37°C. Then the nuclei were stained with DAPI (1:5000, Beyotime Biotechnology, China) for 5 min at room temperature in the dark. The TUNEL-positive cells displaying red nuclear staining were observed and analyzed by a confocal laser-scanning microscope (Leica, Germany). Five fields were chosen randomly under high power magnification, and the apoptosis ratio was calculated as number of TUNEL-positive cells to total number of cells.

### Immunostaining

Cells were plated onto coverslips, handled according to experiment design. The cells were fixed in 4% PFA for 10 min. After washing with PBS three times, fixed cells were permeated with 0.3% Triton X-100 for 10 min, and blocked with 1% bovine serum albumin (BSA) for 1h at room temperature, then incubated overnight at 4°C with primary antibodies: rabbit anti-Nrf2 polyclonal antibody (1:200, Santa Cruz Biotechnology, United States) and rabbit anti-HO-1 polyclonal antibody (1:300, Abcam, United Kingdom). After washing with PBS three times, the cells were incubated with corresponding secondary fluorescent antibodies (1:300, Invitrogen, United States): Alexa Fluor 488 donkey anti-rabbit IgG for 1 h at room temperature. And then the nuclei were counterstained with DAPI (1:5000, Beyotime Biotechnology, China). A confocal laser-scanning microscope (Leica, Germany) was used to observe and analyze fluorescence images.

### Western Blotting Analysis

Cells were plated on 6-well plates, handled according to experiment design and a previous study (Jing et al., 2019). RIPA lysis buffer (Merk Millipore, Germany) with protease inhibitor cocktail (Roche, Swiss) was used to obtain the cell lysate. BCA Protein Assay Kit (Thermo Fisher Scientific, United States) was used to determine the protein concentrations. Each protein sample (30 μg) was loaded for electrophoresis, then transferred onto polyvinylidene difluoride (PVDF) membranes, blocked with 5% non-fat milk and 0.05% Tween-20 at room temperature for 1 h. The membranes were incubated overnight at 4°C with primary antibodies: rabbit anti-Nrf2, mouse anti-β-actin (1:1000, Santa Cruz Biotechnology, United States), rabbit anti-HO-1 (1:2000, Abcam, United Kingdom) and rabbit anti-p-PKCα (1:2000, ABclonal Technology, China). Later, the membranes were washed three times and incubated with proper horseradish peroxidase conjugated secondary antibody for 1 h at room temperature. After washing, the membranes were reacted with enhanced chemiluminescence (ECL) solution (Thermo Fisher Scientific, United States). We used Tanon image system (Shanghai, China) to detect the chemiluminescence signal. The relative intensity of the bands was performed by ImageJ 1.6.0 (NIH, United States).

### Total RNA Extraction and Quantitative Real-Time PCR (RT-PCR) Analysis

Cells were plated on 12-well plates and handled according to experiment design. Total RNA was extracted using Trizol reagent (Invitrogen, Carlsbad, CA, United States) following the manufacturer’s protocol. Reverse transcription and amplifying was carried out using reverse transcriptase and Taq DNA polymerase (Yeasen Biotech Co., Ltd., Shanghai, China), respectively. RT-PCR analyses were performed using the SYBR Green Master Mix Kit (Yeasen Biotech Co., Ltd.) and the PCR thermal cycler (Applied Biosystems, CA, United States). Nrf2, HO-1, NQO1, SOD2, IL-6, IL-10, and TNFα mRNA expressions were determined and quantified to the expression of glyceraldehyde 3-phosphate dehydrogenase (GAPDH). The mRNA relative expressions were normalized to control group. The primer sequences are listed in Table 1.

**TABLE 1:** Primer sequences for qRT-PCR.

**Gene**	**Forward primer/ Reverse primer (5′-3′)**
HO-1	CAAGGAGGTACACATCCAAGCC/ TACAAGGAAGCCATCACCAGCT
NQO1	TGGTGACATAATCCGACAAGAT/ TTACCCACCTGAATGCCATAAT
SOD2	ACGCCACCGAGGAGAAGTACC/ CGCTTGATAGCCTCCAGCAACTC
IL-6	TGGGACTGATGCTGGTGACA/ ACAGGTCTGTTGGGAGTGGT
IL-10	CTGCTATGCTGCCTGCTCTTACTG/ ATGTGGCTCTGGCCGACTGG
TNFα	TGATCGGTCCCAACAAGGA/ TGCTTGGTGGTTTGCTACGA
GAPDH	GATGGTGAAGGTCGGTGTGA/ TGAACTTGCCGTGGGTAGAG

*HO-1, heme oxygenase 1; NQO1, NAD(P)H quinone oxidoreductase 1; SOD2, superoxide dismutase 2; IL-6, interleukin 6; IL-10, interleukin 10; TNFα, tumor necrosis factor α; GAPDH, glyceraldehyde 3-phosphate dehydrogenase.*

### Statistical Analysis

Statistical analysis was performed by SPSS 21.0 (SPSS Inc., Chicago, IL, United States). All data were presented as the mean ± standard error of the mean (SEM) of at least three independent experiments. The average bands density for control groups was set as 1.0, and values of all band density were normalized by the average value of control group to facilitate comparisons. Statistical comparison was performed using Student’s *t*-test or one-way analysis of variance (ANOVA) tests. Statistical significance was deemed at *P* < 0.05.

## Results

### Neurotoxicity Induced by Hemin Led to Primary Astrocytes Apoptosis

**FIGURE 1:**
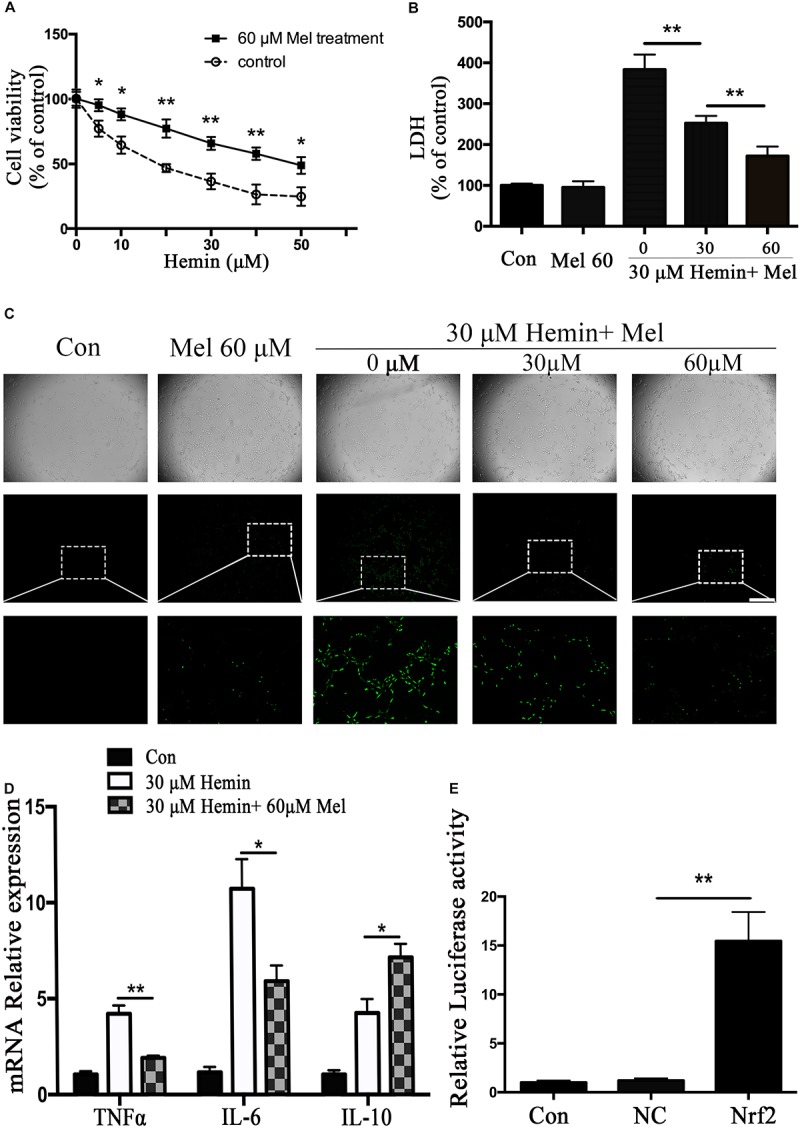
Mel-treatment protected astrocytes from neurotoxicity induced by hemin. **(A)** Astrocytes were exposed to 0, 5, 10, 20, 30, 40, and 50 μM hemin for 24 h with or without 60 μM Mel, then the cell viability was evaluated by CCK-8. **(B)** Astrocytes were exposed to 30 μM hemin with or without 30 or 60 μM Mel for 24 h, and the cell death was evaluated by LDH releasing assay; **(C)** DCFH-DA probes were loaded, the intracellular ROS were observed using fluorescent microscope, bar = 400 μm; **(D)** mRNA expression of TNFα, IL-6, and IL-10 was checked. The relative expression of the mRNA was normalized to control. **(E)** Luciferase activity analysis was examined and normalized to control. The results of densitometric analysis of the bands were plotted into histogram. Difference between groups was analyzed using One-way ANOVA analysis or Student’s *t*-test. ^*^P < 0.05 and ^∗∗^*P* < 0.01.

In order to observe the neurotoxicity induced by hemin on primary astrocytes, CCK-8 and LDH releasing assays were used to evaluate cell viability and cell death. Our results showed that cell viability decreases with hemin dosage (Figure 1A). According to our experimental results, 30 μM hemin was chosen for the subsequent experiments as it could significantly increase cell death (*P* < 0.01).

### Mel Treatment Enhances the Resistance of Astrocytes to Neurotoxicity From Hemin, and Regulated Cytokines mRNA Expression

**FIGURE 2:**
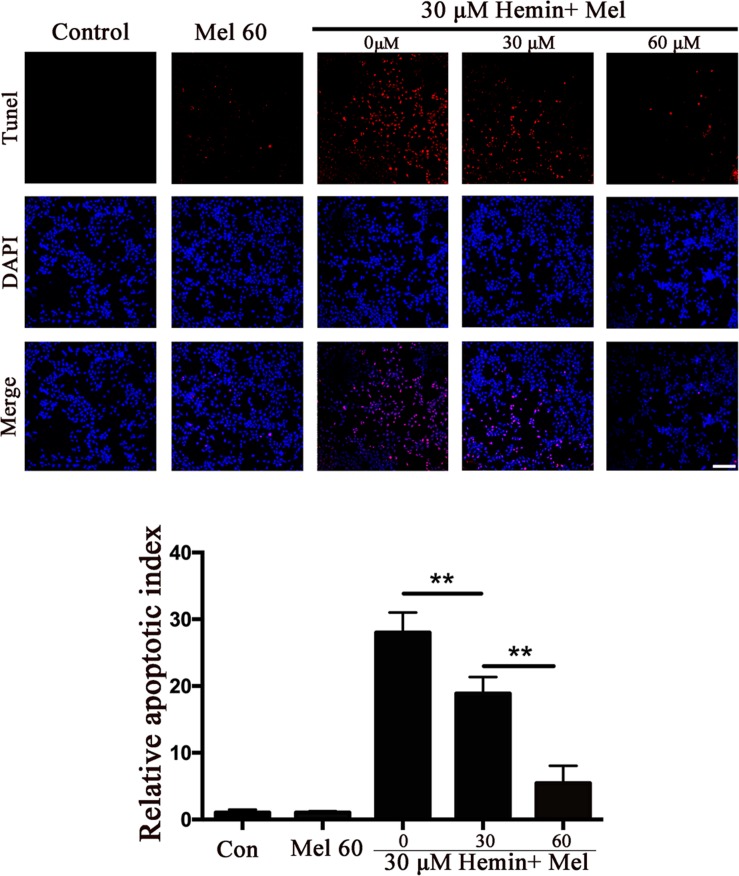
The effect of Mel on astrocytes apoptotic ratio in five groups was indicated. TUNEL staining (red) was used to mark apoptotic cells, bar = 200 μm. The apoptosis rate was plotted into histogram and the relative expression of the proteins was normalized to control. Difference between groups was analyzed using One-way ANOVA analysis or Student’s *t*-test. ^∗∗^*P* < 0.01.

To explore whether Mel-treated astrocytes gain resistance to neurotoxicity from hemin, primary astrocytes were exposed to 30 μM hemin for 24 h, with or without 30 or 60 μM Mel. After Mel administration, astrocytes were resistant to neurotoxicity induced by hemin (Figure 1A) and LDH releasing assay (Figure 1B) showed Mel-treatment significantly decreased LDH releasing. The cell apoptosis rate decreased significantly in Mel-treated astrocytes compared to non-treated cells (*P* < 0.001) (Figure 2). We found that the protective effect of Mel was dose-dependent. Higher Mel doses presented a stronger antitoxic effect than lower doses (Figures 1A,B). Then we checked the mRNA expression of cytokines. TNFα and IL-6 decreased significantly after Mel treatment (*P* < 0.001 and *P* < 0.05, respectively), while IL-10 increased significantly (*P* < 0.05) (Figure 1D).

### Mel Down-Regulated ROS Accumulation Induced by Hemin

The neurotoxicity of hemin is mainly due to the production of ROS. To explore whether Mel-treatment could protect astrocytes against neurotoxicity from hemin by blocking intracellular ROS accumulation, ROS probe DCFH-DA was loaded into the cells. The results (Figure 1C) showed that hemin could significantly increase ROS accumulation while Mel-treatment significantly reduced intracellular ROS accumulation induced by hemin (*P* < 0.05).

### Mel Up-Regulated Astrocytes HO-1 Expression After Hemin Exposure, and Up-Regulated NQO1, SOD2 mRNA Expression Simultaneously

**FIGURE 3:**
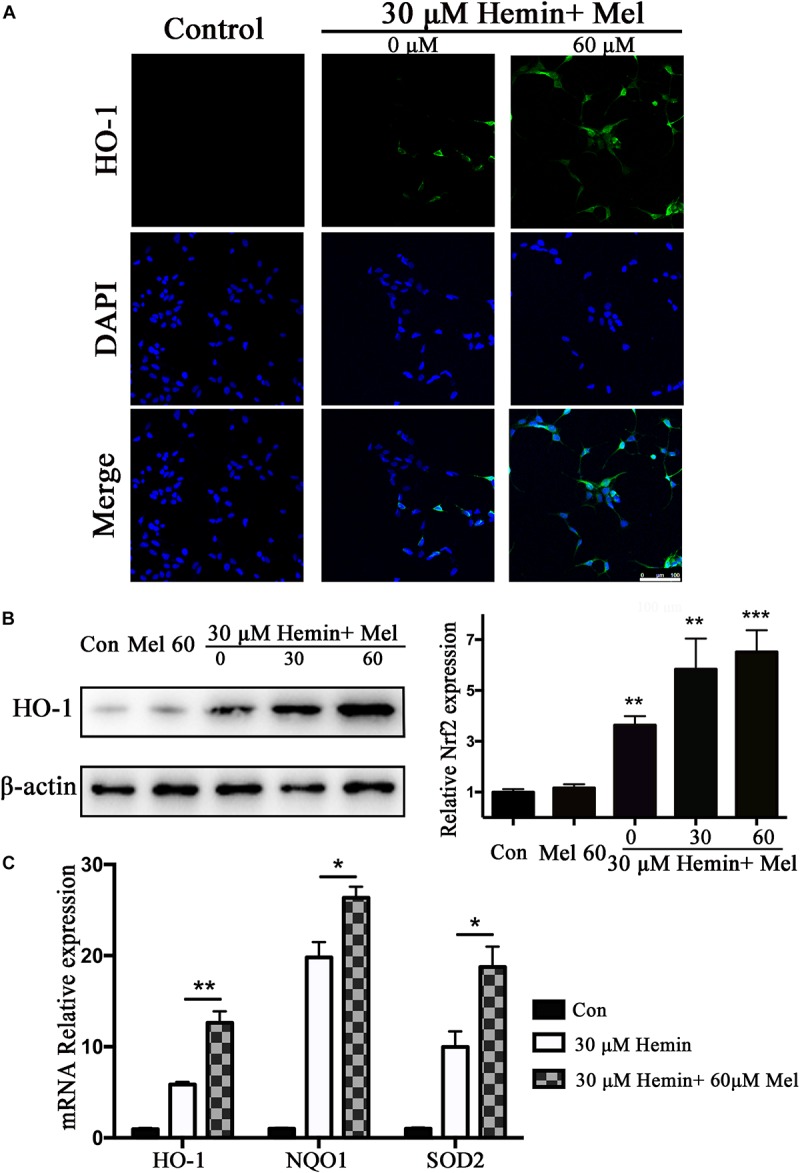
Mel-treatment induced HO-1 expression in astrocytes. **(A)** Immunostaining showed the expression of HO-1 in astrocytes treated with or without 60 μM Mel for 24 h, the nuclei were counterstained with DAPI, bar = 100 μm. **(B)** Western blotting analysis of HO-1 expression in astrocytes treated with Mel of indicated dose. **(C)** mRNA expression of HO-1, NQO1, and SOD2 in astrocytes treated with Mel was further examined. The relative expression of the proteins and mRNA was normalized to control. The results of densitometric analysis of the bands were plotted into histogram. Difference between groups was analyzed using One-way ANOVA analysis or Student’s *t*-test. ^*^P < 0.05, ^∗∗^*P* < 0.01, and ^∗∗∗^*P* < 0.001 vs. control group.

HO-1 catalyzes hemin oxidative catabolism. In order to explore whether the protective effect of Mel-treatment is related to HO-1 induction, the expression of HO-1 was detected by immunostaining and western blotting. The immunostaining revealed that the HO-1 staining in astrocytes after hemin exposure increased after Mel-treatment (Figure 3A). Then we further investigated HO-1 protein expression by western blotting analysis. The results revealed that the HO-1 protein expression after Mel treatment was increased in parallel with HO-1 immunostaining (*P* < 0.01) (Figure 3B). The up-regulation capability of Mel was dose-dependent.

Subsequently, we further examined the effect of Mel on the mRNA expression of phase II antioxidant enzymes besides HO-1. The results were consistent with the protein expression of HO-1 (*P* < 0.01, *P* < 0.01, and *P* < 0.01, respectively) (Figure 3C).

### Mel Increased Astrocytes Nrf2 Expression and Promoted Nrf2 Nuclear Translocation

**FIGURE 4:**
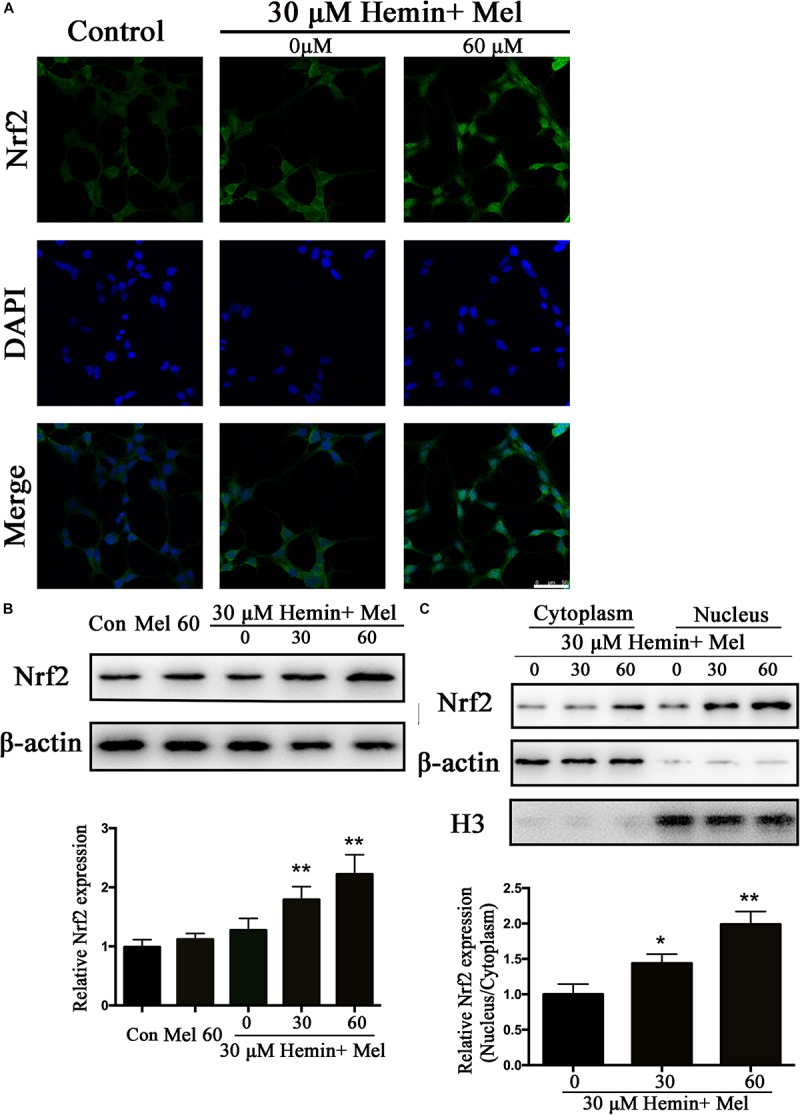
Mel-treatment induced Nrf2 expression and promoted its nuclear translocation. **(A)** Immunostaining showing the subcellular expression of Nrf2 in astrocytes treated with or without 60 μM Mel for 24 h. The nuclei were counterstained with DAPI, bar = 50 μm. **(B)** Western bloting analysis of Nrf2 expression in astrocytes treated with Mel of indicated dose. **(C)** Nrf2 protein expression of cytoplasmic and nuclear from 0, 30, 60 μM Mel treated was analyzed. β-actin and H3 were, respectively, used as loading control for cytoplasmic and nuclear protein expression. The relative expression of the proteins was normalized to control. The results of densitometric analysis of the bands were plotted into histogram. Difference between groups was analyzed using One-way ANOVA analysis or Student’s *t*-test. ^*^P < 0.05 and ^∗∗^*P* < 0.01 vs. control group or 0 μM group.

Nrf2 is the major endogenous regulator of antioxidant reaction. Luciferase gene reporter showed that the fluorescence activity of astrocytes with overexpressed Nrf2 plasmid increased significantly, compared with the NC plasmid (*P* < 0.01) (Figure 1E). By immunostaining and western blotting, we observed whether the HO-1 expression was regulated by Nrf2 signaling pathway. The immunostaining (Figure 4A) showed that activated Nrf2 transferred into the nuclei of astrocytes under the stimulation of Mel. The protein expression of Nrf2 after 30 and 60 μM Mel stimulation (Figure 4B) was higher than those of no-treatment groups (*P* < 0.01). Western blotting of nuclear and cytoplasmic samples (Figure 4C) revealed that the Nrf2 ratio of nucleus to cytoplasm of astrocytes treated with Mel was significantly higher than that of no-treatment group (*P* < 0.01) and Nrf2 nuclear augmentation also showed a dose dependence (Figure 4C).

### Mel Increased Astrocytes p-PKCα Expression, and Nrf2 and HO1 Expression Decreased After PKC Inhibition

**FIGURE 5:**
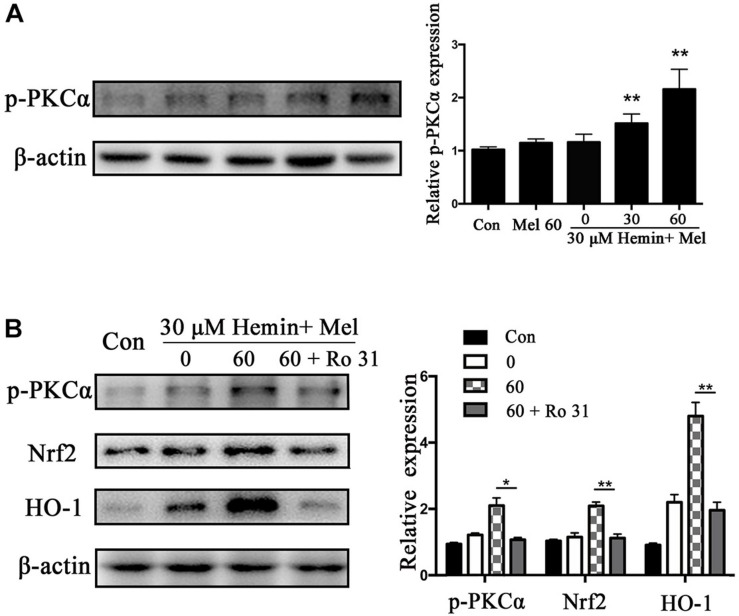
P-PKCα expression in astrocytes exposed to hemin with Mel treatment, with or without PKC inhibitors. Western blotting analysis of p-PKCα expression in astrocytes treated with Mel of indicated dose **(A)**. **(B)** After administration of PKC inhibitors (RO 31), p-PKCα and its downstream expression were significantly inhibited. The relative expression of the proteins was normalized to control. The results of densitometric analysis of the bands were plotted into histogram. Difference between groups was analyzed using One-way ANOVA analysis or Student’s *t*-test. ^*^P < 0.05 and ^∗∗^*P* < 0.01 vs. control group.

Protein kinase C activation is a key event of Nrf2 nuclear translocation during oxidative stress. We observed that p-PKCα protein expression was higher in the Mel treated group than in untreated ones (*P* < 0.01) (Figure 5A), with a simultaneous increase in Nrf2 and HO1 expression. When we administrated the PKC inhibitor (Ro 31) in addition to Mel, both p-PKCα (*P* < 0.05) and HO-1 and Nrf2 (*P* < 0.01 and *P* < 0.01, respectively) upregulations by Mel were suppressed (Figure 5B).

### Luz Inhibits the Protective Effect of Mel and Down-Regulated HO-1, Nrf2, and p-PKC Expression of Mel-Treated Astrocytes After Hemin Exposure

**FIGURE 6:**
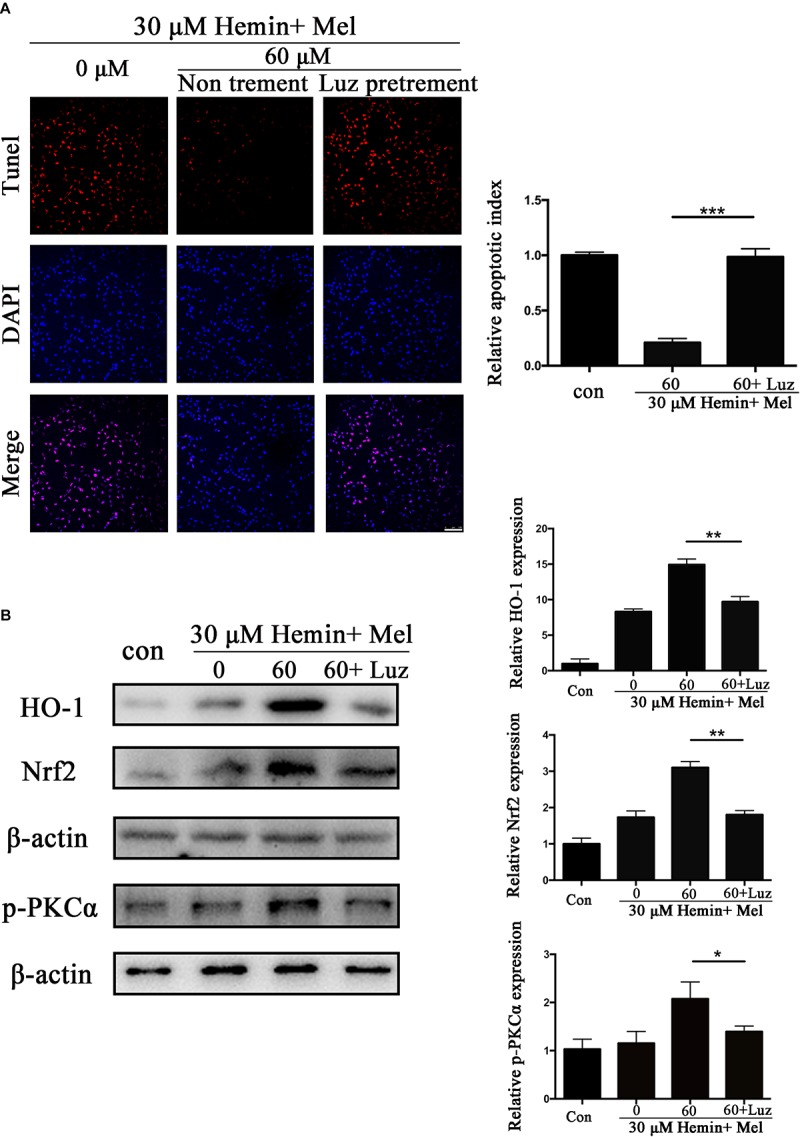
Luz inhibited the protection effect of Mel and suppressed HO-1 and Nrf2 expression after hemin exposure. Astrocytes were transfected with or without Luz for 6 h, then followed by 30 μM hemin incubation, with or without 60 μM Mel for 24 h, **(A)** TUNEL staining (red) was used to mark apoptotic cells, bar = 100 μm. **(B)** Western bloting analysis of p-PKCα, Nrf2, and HO-1 protein expression was examined according to the experimental design. The relative expression was normalized to control. The results of apoptosis rate and densitometric analysis of the bands were plotted into histogram. Difference between groups was analyzed using One-way ANOVA analysis or Student’s *t*-test. ^*^P < 0.05, ^∗∗^*P* < 0.01, and ^∗∗∗^*P* < 0.01.

To explore the potential mechanisms of Mel, we administrated Mel receptor inhibitor (Luz) in addition to Mel treatment. TUNEL staining (Figure 6A) showed that after Luz administration, the numbers of TUNEL-positive cells were significantly increased than in the Mel group (*P* < 0.001). Luz also strongly suppressed the protein expression of HO-1, Nrf2, and p-PKCα up-regulated by Mel compared to Mel group (*P* < 0.01, *P* < 0.01, and *P* < 0.05, respectively) (Figure 6B).

### Nrf2 Knockdown Offset the Protection Effect of Mel on Neurotoxicity From Hemin

**FIGURE 7:**
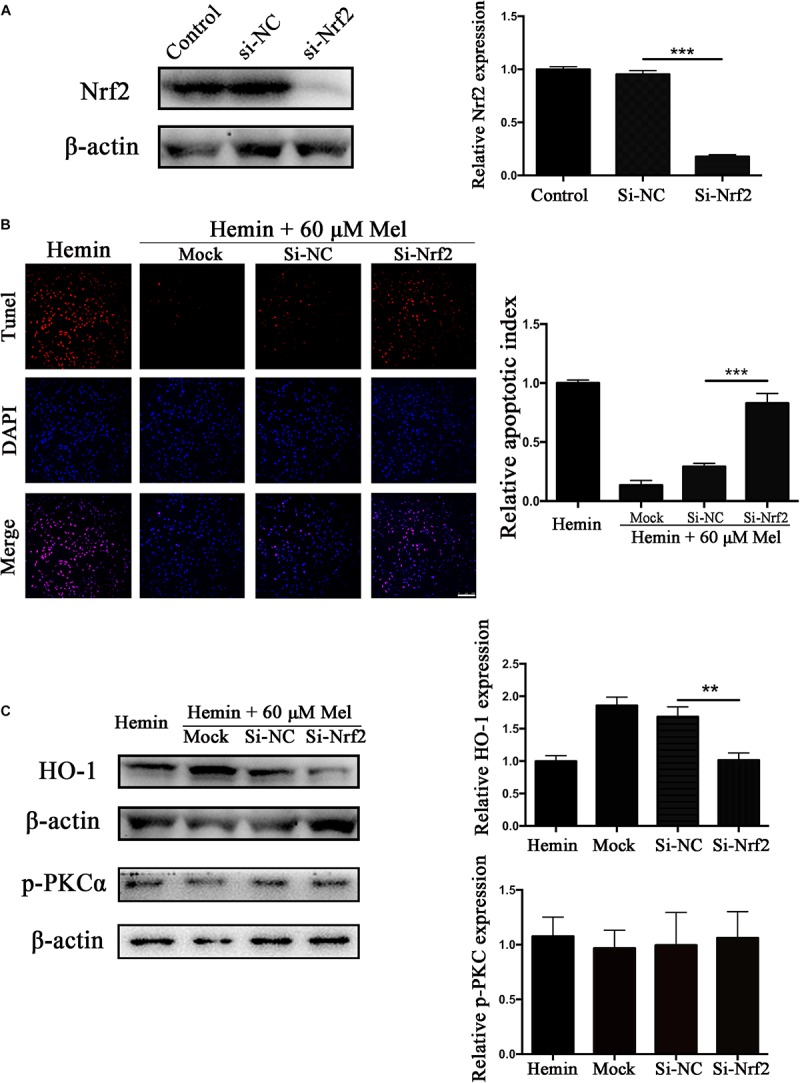
Nrf2 knockdown suppressed Mel-induced upregulation of HO-1 expression and increased the numbers of TUNEL-positive cells. **(A)** Western blotting analysis of Nrf2 expression in control, si-NC, si-Nrf2 transfected astrocytes. Astrocytes were transfected with or without si-NC or si-Nrf2 for 48 h, then followed by 30 μM hemin incubation, with or without 60 μM Mel for 24 h; **(B)** TUNEL staining (red) was used to mark apoptotic cells, bar = 100 μm; **(C)** The HO-1 and p-PKCα expression was analyzed by western blotting. The relative expression was normalized to control. The results of apoptosis rate and densitometric analysis of the bands were plotted into histogram. Difference between groups was analyzed using One-way ANOVA analysis or Student’s *t*-test. ^∗∗^*P* < 0.01 and ^∗∗∗^*P* < 0.01.

To explore whether the protection effect of Mel treatment relied on Nrf2, we transfected astrocytes with si-Nrf2 and si-NC. We used western blotting to check the efficiency of knockout, and about 82.14% of Nrf2 expression was restrained by si-Nrf2 (*P* < 0.001) (Figure 7A). After astrocytes infected with si-Nrf2 and si-NC were treated with 30 μM hemin for 24 h, with or without 60 μM Mel, TUNEL staining (Figure 7B) revealed that si-Nrf2 significantly increased the number of TUNEL-positive cells compared to si-NC (*P* < 0.001). Western blotting showed that si-Nrf2 significantly depressed HO-1 up-regulation induced by Mel, compared to si-NC (*P* < 0.01), but there was no significant difference in p-PKC (Figure 7C).

## Discussion

The major findings of this study are as below: (1) the cell viability of astrocytes was decreased after hemin exposure, in a dose-dependent manner; (2) astrocytes are extensively damaged by neurotoxicity induced by hemin without Mel treatment, but after treated with Mel, Mel helped astrocytes resist the neurotoxicity and reduce the degree of damage; (3) Mel administration induced PKCα phosphorylation, Nrf2 upregulation and nuclear translocation in astrocytes, and led to phase II enzyme HO-1 upregulation; (4) Nrf2 and HO1 protein expression upregulated by Mel were blocked after administration of PKC inhibitor, Ro 31; (5) Mel-induced activation of PKCα/Nrf2/HO1 pathway could be partly abolished by Mel receptor inhibitor, Luz; (6) the *in vitro* protective effect of Mel on astrocytes was PKCα/Nrf2 dependent.

Mel, secreted by the pineal gland, possesses multiple pharmacological properties (Cipolla-Neto and Amaral, 2018). Mel as well as its metabolites are highly effective endogenous antioxidants. They are often used as a protective factor and antioxidant in many experiments and studies (Reiter et al., 2000; Cipolla-Neto and Amaral, 2018). Several recent studies propose that Mel prevents kidney injury (Sener et al., 2002), pancreatitis injury (Jung et al., 2010), and liver injury (Jung et al., 2009; Kang and Lee, 2012) by decreasing oxidative stress. In terms of neuroprotection, Mel has been reported to play an active role in several neurological disease, such as epilepsy (Brigo et al., 2016), Parkinson’s disease (Mendivil-Perez et al., 2017), cerebral ischemia (Yang et al., 2015), intracerebral Hemorrhage (Wang Z. et al., 2018), and SAH (Dong et al., 2016; Zhao et al., 2017).

These beneficial properties impel us to think over the mechanism of Mel on astrocytes to protect ICH from oxidative stress. Both Mel and Nrf2 pathways play a vital role in oxidative stress. To date, although several studies have reported about the role of Mel on the Nrf2/ARE pathway (Jung et al., 2009, 2010; Negi et al., 2011; Wang et al., 2012; Deng et al., 2015; Kleszczynski et al., 2016; Trivedi et al., 2016; Cao et al., 2017), the mechanism is still not definitely clear. Our results were in favor of neuroprotection of Mel on astrocytes as Mel treatment not only reduced ROS accumulation but also enhances the resistance of astrocytes to neurotoxicity from hemin *in vitro*. Furthermore, we found that Mel treatment increased PKCα phosphorylation and Nrf2 and its phase II enzyme HO1 expression when compared to the untreated group, being those effects dose-dependent, which impelled us to consider Mel as a potential neuroprotection drug against ICH through PKCα/Nrf2/HO1 pathway.

Protein kinase C is one of several protein kinases able to modify Nrf2 to activate its release from keap1 (Huang et al., 2002). PKC phosphorylation of Nrf2 serine 40 results in the escape or release of Nrf2 from Keap1, translocate to the nucleus, and bind to the ARE that leads to coordinated activation of gene expression (Huang et al., 2002; Niture et al., 2010). It was reported that direct phosphorylation of Nrf2 by PKC is a key event of Nrf2 nuclear translocation in oxidative stress (Huang et al., 2000). In addition, PKCα inhibitors could reduce the expression of Nrf2, leading to the down-regulation of HO-1 (Yun et al., 2010). Nrf2 is an important transcription factor regulating antioxidant defense (Deng et al., 2015; Kleszczynski et al., 2016). Once stimulated by oxidative stress, Nrf2 is released by Keap1 and would be translocated to the nucleus binding to ARE and promoting the transcription of HO-1, phase II detoxification enzyme genes (Wang et al., 2012; Chen et al., 2015; Deng et al., 2015; Liu et al., 2015; Santofimia-Castano et al., 2015; Kleszczynski et al., 2016). Nrf2-ARE pathway is considered as a multi-organ protective agent and has been reported to play a key role in several CNS diseases, such as SAH (Chen et al., 2011), cerebral ischemia (Shih et al., 2005), traumatic brain injury (Wang J. et al., 2018), and cerebral hemorrhage (Chen et al., 2015). In addition, Nrf2 signaling pathway would be activated in astrocytes to protect astrocytes as well as their adjacent neurons from oxidative damage (Kraft et al., 2004; Wang J. et al., 2018). Wang et al. (2007) also pointed out that when the Nrf2 gene was knocked out, neurological function might be impaired after the ICH. The mechanism may relate to ROS-induced DNA damage and neuronal cell death by apoptosis (Ito et al., 2001; Wang and Tsirka, 2005; Wang et al., 2007). Our results were parallel with those reports and indicated that phosphorylation of PKCα increased after MEL treatment, followed by up-regulation of Nrf2 and HO-1, which subsequently led to a decrease in ROS accumulation and apoptosis after hemin exposure.

Subcellular distribution of Nrf2 was further studied. The results indicated that Nrf2 expression was upregulated and Nrf2 translocated into the nucleus after Mel treatment. It was reported by Negi et al. (2011) that Mel stabilizes and activates Nrf2 in both cytoplasm and nucleus. In our study, Mel did increase Nrf2 expression both in nucleus and cytoplasm, but the growth increase in nucleus was more significant than that in cytoplasm. When the cellular protective mechanism was activated by stress impacts, Nrf2 will be translocated into the nucleus, which may be an effective way to maintain cell survival (Kleszczynski et al., 2016). This may explain our results that Nrf2 expression in nucleus was higher than that in cytoplasm. We also found that the Nrf2 upregulation and nuclear translocation depended on the phosphorylation of PKCα, and this phenomenon was terminated by PKC inhibitors. When we used Mel receptor inhibitors, Luz, these positive results were blocked, phosphorylation of PKCα was inhibited, upregulation of Nrf2 and HO1 were reversed, and correspondingly, nuclear translocation was suppressed, which confirmed that Mel protects astrocytes against apoptosis through PKCα/Nrf2-HO1 signaling pathway.

To further confirm that the protective effect of Mel treatment is Nrf2 dependent, we knocked down the expression of Nrf2 with Nrf2 specific siRNA. Our results indicated that Mel-induced HO-1upregulation was significantly suppressed by si-Nrf2, and the ROS accumulation and cell apoptosis were significantly increased, compared to si-NC group. Rodriguez et al. (2007) have shown that disruption of Nrf2 enhanced the upregulation of NF-κB activity and pro-inflammatory cytokines in brain injury, and vice versa, a low level of TNFα (2–5 ng/ml) could evoke significant nuclear translocation of Nrf2 with increased DNA/promoter binding and transactivation of Nrf2 targets (Shanmugam et al., 2016). Such phenomenon indicated that there might be an interaction between the pro-inflammatory signaling pathway and the Nrf2/HO-1 signaling pathway (Kleszczynski et al., 2016). Yin et al. (2015) have shown that the HO-1 inhibitor, ZPP-IX, not only decreased the HO-1 expression and inhibited the Nrf2 entering nucleus, but also triggered the NF-κB entering nucleus, resulted in the over-expression of NF-κB and TNF-α. This result corresponded to the research of Poss and Tonegawa (1997) that animals developed a chronic inflammatory disease in a HO-1 knockout mice model. Our study also showed that MEL therapy downregulated TNFα and IL-6 expression, and upregulated IL-10 expression in astrocytes after hemin exposure. This was probably due to the antioxidant defense mechanism induced by elevated Nrf2 and nuclear translocation, or additional activation of inflammatory pathways, which need to be further explored in our future research.

**FIGURE 8:**
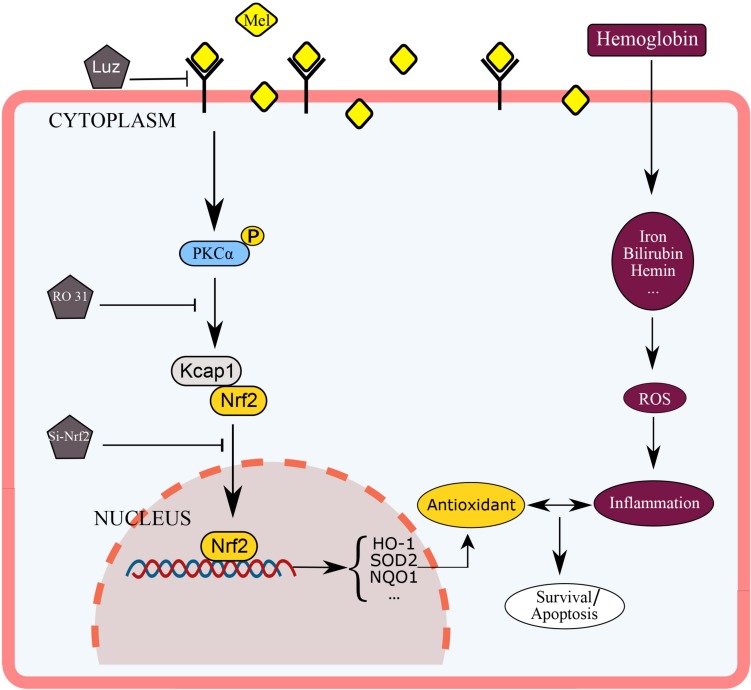
Diagram outlining the mechanism of Mel on PKCα/Nrf2/HO-1 signaling pathways. Mel induced PKCα phosphorylation (p-PKC), nuclear translocation of Nrf2 in astrocytes, and upregulation of HO-1, then restraining ROS accumulation and cell apoptosis.

This experiment used mice astrocytes to simulate oxidative stress of cerebral hemorrhage *in vitro* in order to study the protective effect of Mel on astrocyte through PKCα/Nrf2/HO-1 pathway. As far as we know, this may be the first report that shows that melatonin attenuates hemin induced oxidative damage in primary astrocytes via PKCα/Nrf2/HO1 signaling pathway *in vitro*. The potential mechanism of Mel on PKCα/Nrf2/HO-1 signaling pathways is shown in Figure 8. Unfortunately, there were still several limits in our studies. We only used *in vitro* Nrf2 knockout model and PKC inhibition to verify the effect of Mel on PKCα/Nrf2/HO-1 pathway. We lack Nrf2 knockout and PKC inhibition experiments *in vivo* to verify our theory, there may be other possibilities that Mel affects PKCα/Nrf2/HO1 pathways through other independent effects, such as cross-transmission between signaling pathways, microenvironment effects and cell-to-cell connections. It is important to note that Luz is just a Mel receptor inhibitor. The inhibition of the membrane receptors influence many of Mel actions, but does not inhibit all of them, as Mel presents direct actions, that are non-receptor dependent, and nuclear receptor-dependent actions. This study deals with the inhibition of the membrane receptor of Mel, so we set up blank group, control group and so on to minimize the error. In further research, we will focus on the protective effect of Mel on ICH through PKCα/Nrf2/HO1 signaling pathway *in vivo*.

Our results suggest that Mel activated PKCα/Nrf2/HO1 signaling pathway, inducing PKCα phosphorylation, upregulation as well as nuclear translocation of Nrf2, to protect astrocytes against neurotoxicity, and apoptosis from hemin. The protective effect of Mel on astrocyte depends on PKCα phosphorylation and the activation of Nrf2. The mechanisms by which Mel is coupled to PKCα and Nrf2 deserve future study. It is still worthwhile to take PKCα/Nrf2/HO1 signaling pathway combined with Mel as a target for neuroprotection after ICH.

## Data Availability

The raw data supporting the conclusions of this manuscript will be made available by the authors, without undue reservation, to any qualified researcher.

## Ethics Statement

This study was carried out in accordance with the recommendations of “Evidence of the Animal Experimental Ethics Committee of Ruijin Hospital, Shanghai Jiao Tong University School of Medicine.” The protocol was approved by the “Laboratory Animal Ethics Committee of Ruijin Hospital, Shanghai Jiao Tong University School of Medicine.”

## Author Contributions

XC, QS, and LB designed the research and wrote the manuscript. XC and ZX analyzed the results. HL prepared and completed the astrocytes isolation and culture. HL and ZZ collected the data and carried out the statistical analysis. YS, BW, and LB provided useful suggestions on the experiment design and reviewed the manuscript. YS and LB provided funds collection. QS and LB assisted with reviewing, editing the manuscript, and provided expertize and feedback.

## Conflict of Interest Statement

The authors declare that the research was conducted in the absence of any commercial or financial relationships that could be construed as a potential conflict of interest.
